# Cytotoxic, Genotoxic and Senolytic Potential of Native and Micellar Curcumin

**DOI:** 10.3390/nu13072385

**Published:** 2021-07-13

**Authors:** Lea Beltzig, Anna Frumkina, Christian Schwarzenbach, Bernd Kaina

**Affiliations:** Institute of Toxicology, University Medical Center, Obere Zahlbacher Straße 67, 55131 Mainz, Germany; Lea.beltzig@uni-mainz.de (L.B.); frumkian@uni-mainz.de (A.F.); schwarzenbach@uni-mainz.de (C.S.)

**Keywords:** curcumin, bioavailability, micelles, apoptosis, comet assay, genotoxicity, senolytics

## Abstract

Background: Curcumin, a natural polyphenol and the principal bioactive compound in *Curcuma longa*, was reported to have anti-inflammatory, anti-cancer, anti-diabetic and anti-rheumatic activity. Curcumin is not only considered for preventive, but also for therapeutic, purposes in cancer therapy, which requires a killing effect on cancer cells. A drawback, however, is the low bioavailability of curcumin due to its insolubility in water. To circumvent this limitation, curcumin was administered in different water-soluble formulations, including liposomes or embedded into nanoscaled micelles. The high uptake rate of micellar curcumin makes it attractive also for cancer therapeutic strategies. Native curcumin solubilised in organic solvent was previously shown to be cytotoxic and bears a genotoxic potential. Corresponding studies with micellar curcumin are lacking. Methods: We compared the cytotoxic and genotoxic activity of native curcumin solubilised in ethanol (Cur-E) with curcumin embedded in micells (Cur-M). We measured cell death by MTT assays, apoptosis, necrosis by flow cytometry, senolysis by MTT and C12FDG and genotoxicity by FPG-alkaline and neutral singe-cell gel electrophoresis (comet assay). Results: Using a variety of primary and established cell lines, we show that Cur-E and Cur-M reduce the viability in all cell types in the same dose range. Cur-E and Cur-M induced dose-dependently apoptosis, but did not exhibit senolytic activity. In the cytotoxic dose range, Cur-E and Cur-M were positive in the alkaline and the neutral comet assay. Genotoxic effects vanished upon removal of curcumin, indicating efficient and complete repair of DNA damage. For inducing cell death, which was measured 48 h after the onset of treatment, permanent exposure was required while 60 min pulse-treatment was ineffective. In all assays, Cur-E and Cur-M were equally active, and the concentration above which significant cytotoxic and genotoxic effects were observed was 10 µM. Micelles not containing curcumin were completely inactive. Conclusions: The data show that micellar curcumin has the same cytotoxicity and genotoxicity profile as native curcumin. The effective concentration on different cell lines, including primary cells, was far above the curcumin concentration that can be achieved systemically in vivo, which leads us to conclude that native curcumin and curcumin administered as food supplement in a micellar formulation at the ADI level are not cytotoxic/genotoxic, indicating a wide margin of safety.

## 1. Introduction

Considerable interest has been addressed to natural compounds that have a health-beneficial potential. Plants are especially rich in bioactive natural compounds [1,2]. In particular, plant (poly)phenols [3] were reported to have a high therapeutic and preventive potential, combined with negligible adverse effects [4,5]. One of these compounds is curcumin (1,7-bis (4-hydroxy-3-methoxyphenyl)-1,6-heptadiene-3,5-dione), a naturally occurring polyphenol present in the rhizome of *Curcuma longa* L. and other curcuma species [6]. Curcuma, which contains a mixture of curcuminoids of which curcumin is the main component, has a long tradition as a food supplement in Asia. Moreover, it is being used in traditional Chinese medicine for the treatment of a wide variety of diseases, including inflammation-related disorders and neurodegenerative diseases [7]. Whether the curcuma-based traditional Chinese medicine formulae are effective in the treatment of diseases is a matter of dispute [8].

With the identification of curcumin as the active constituent of curcuma, numerous molecular-biological studies have been performed, and several molecular targets have been identified, including NF-kB, MAP-kinase, p53, NRF2, AKT, COX-2 and EGFR [9,10,11]. Some of these targets are key nodes in inflammation and cancer, supporting the rationale that curcumin may be beneficial in cancer prevention. Clinical studies with curcumin point to its anti-rheumatic, anti-cancer, anti-diabetic and wound-healing potential [12,13,14].

A drawback in the use of curcumin, and a major point of criticism regarding its health effects, is the low bioavailability of the polyphenol [15,16] due to its low water solubility and rapid metabolism [17,18]. Thus, curcumin administered in solid form cannot be taken up systemically, and solubilization in alkaline media results in rapid decomposition of the compound [19]. Since the low bioavailability clearly hampers the application of curcumin as a bioactive agent, several strategies have been pursued to overcome these limitations, including the use of adjuvants for inhibiting its metabolism, micronization, binding to cyclodextrin and embedding it into liposomes (for a review, see [20]). The incorporation of curcumin into micelles was found to be the hitherto most successful strategy to enhance its oral bioavailability, resulting in reasonable blood plasma levels [21,22,23], and even significant concentrations were found in brain tumors of patients ingesting micellar curcumin prior to tumor resection [24].

In view of the widespread use of curcumin as a food, the question of a possible genotoxic effect deserves particular attention. Curcumin does not bind to DNA and does not form adducts. It does not bear mutagenic activity, as confirmed in the Ames test [25]. In mammalian cells, the effects of curcumin are complex. Thus, curcumin is an anti-oxidant [26], and therefore it can exhibit protective effects by weakening the activity of ROS-generating genotoxins, which explains the protection against radiation-induced DNA damage [27]. However, in the high (micromolar) dose range, curcumin was reported to act as a radical generator and pro-oxidant: it causes a ROS burst and concomitantly oxidative DNA damage. Thus, it has been shown that ROS production increased progressively at curcumin exposure concentrations of 10–100 µM [28], and curcumin induces 8-oxo-guanine in HepG2 cells at concentrations >2.5 µg/mL (6.8 µM) [29]. In the same concentration range (2.5–10 µg/mL), curcumin induced micronuclei in rat PC12 cells [30]. Based on these data, curcumin administered in high (cytotoxic) doses can be regarded as a substance with a weak genotoxic potential. Curcumin was also shown to induce cellular senescence [31,32].

Here, we used the micellar formulation of curcumin (Cur-M), which was previously shown to enhance significantly the curcumin bioavailability in humans [21] and compared it with curcumin solubilised in ethanol (Cur-E) as to its cytotoxic and genotoxic potential. We measured dose dependently viability, apoptosis, necrosis and genotoxic effects using the FPG-alkaline and neutral comet assays. We also assessed the senolytic activity of Cur-M, i.e., the ability to kill specifically senescent cells. We show that curcumin induces cyto- and genotoxicity in a narrow, high dose range between 10 and 60 µM. There was no difference between Cur-E and Cur-M and between cancer and normal cells. Genotoxic effects vanished upon post-incubation in the absence of curcumin, indicating they are transient and subject to repair. Micelles not containing curcumin were negative in all assays, i.e., they do not bear a cytotoxic or genotoxic potential. Curcumin was not senolytic. Overall, the data show that the increased bioavailability of micellar curcumin is not linked to a higher cytotoxic and genotoxic potential compared to native curcumin administered in an organic solvent.

## 2. Materials and Methods

### 2.1. Reagents

Native curcumin (Diferuloylmethan) from *Curcuma longa* (Turmeric) was purchased from Sigma-Aldrich, Germany (CAS 458-37-7) and was dissolved in ethanol at a stock concentration of 10 mM. Micellar curcumin (NovaSol containing highly purified curcumin, ≥95%; Kancor Mane, Kerala, India) and control micells containing water were produced on Tween-80 basis and kindly provided by AQUANOVA AG (Darmstadt, Germany). The micellar stocks were always freshly prepared by weighting and dilution in PBS of a given amount of micells, giving a stock solution of 10 mM. Stocks were stored in the dark at 4 °C for no more than 3 days. Temozolomide (TMZ) was a gift from Dr. G. Margison (Manchester, UK) and was handled as described [33]. For other chemicals and reagents, see the corresponding method section.

### 2.2. Cell Lines and Culture Conditions

The primary human cell lines HUVEC, HUASMC and hPC-PL were purchased from PromoCell. EA.hy926, a cell line created by fusion of the A149 human lung carcinoma and the HUVEC cell line, and the human glioblastoma lines LN229 and A172, were purchased from American Type Culture Collection (ATCC). The human fibroblast cell line VH10T is a diploid telomerase-immortalized line, which was a kind gift from Prof. L. Mullenders, Leiden. The primary cell lines were cultured in endothelial cell growth medium 2, smooth muscle cell growth medium 2 and pericyte growth medium, purchased from PromoCell (Heidelberg, Germany), respectively. EA.hy926, LN229 and A172 were cultured in DMEM or DMEM GlutaMax (Gibco, Life Technologies Corporation, Paisley, UK) supplemented with 10% fetal calf serum (FCS; Gibco, Life Technologies Corporation, Paisley, UK), and for EA.hy926 with 1% HAT. All cells were maintained at 37 °C in a humidified atmosphere containing 5% CO_2_. To ensure exponential growth during the whole experiment period, cells were seeded 48 h prior to treatment, and cell densities were chosen accordingly.

### 2.3. MTT Assay

Cells were seeded in 96-well plates and 2 days later treated with curcumin dissolved in ethanol or packed in micelles for 48 h. For the thiazolyl blue tetrazolium bromide (MTT) assay, cells were washed with PBS, and incubated for 2 h with 100 µL DMEM without phenol red containing 0.5 mg/mL MTT reagent (BIOMOL, Hamburg, Germany). The staining solution was carefully removed, 100 µM 0.04 N HCL were added to each well, and plates were incubated on a shaker (200 rpm) at room temperature for 10 min. Plates were measured in triplets using the Berthold microplate reader at OD_570_ and expressed as absorbance relative to the non-treated control.

### 2.4. Apoptosis and Necrosis

The amount of apoptotic and necrotic cells was measured by flow cytometry using annexin V (AV) and propidium iodine (PI) staining as described [34]. In brief, cells, including the supernatants, were collected, centrifuged and stored on ice. They were incubated for 15 min at RT in 50 µL annexin binding buffer (10 mM Hepes, 140 mM NaCl, 25 µM CaCl_2_) containing 2.5 µL AV/FITC (Miltenyi Biotec GmbH, Bergisch Gladbach, Germany). For PI staining, 10 µL PI from a 50 µg/mL stock solution (Sigma-Aldrich, Steinheim, Germany) were added to each sample. Cells were kept in the dark until measurement. Before data acquisition using the FACS Canto II flow cytometer (Becton Dickinson GmbH, Heidelberg, Germany), cells were diluted in an adjusted amount of annexin binding buffer. Data were analysed using the Flowing Software 2 (Perttu Terho, Turku Center for Biotechnology, University of Turku, Finland). Apoptotic cells were defined as AV+/PI− cells, late apoptosis AV+/PI+ and necrosis as AV−/PI+. A very low amount of AV−/PI+ cells were found in all our assays. To make sure that necrosis was not completely neglected, we classified the AV+/PI+ population as late apoptosis/necrosis (see Supplementary Materials, Figure S1).

### 2.5. Senescence

Temozolomide-induced senescence was measured via flow cytometry using C12FDG staining. Immediately before harvest, cells were treated with 300 µM chloroquine for 30 min to reduce endogenous SA-β-galactosidase activity. Thereafter, C12FDG was added (33 µM), and cells were incubated for an additional 90 min. Cells were washed and resuspended in PBS for measurement, while cells were kept on ice in the dark.

### 2.6. Determination of Senolytic Activity

Senescent cells were obtained by treatment of glioblastoma cancer cells (LN229 and A172) with temozolomide. To this end, 1.5 × 10^5^ exponentially growing cells were trypsinized, seeded per 10 cm dish and treated 2 days later with 50 µM temozolomide. After 8–10 days, the population contained about 80% senescent cells, determined by SA-ßGAL cytochemistry and C12FDG flow cytometry essentially as reported previously [34]. Measurement occurred in a FACS Canto II flow cytometer (Becton Dickinson GmbH, Heidelberg, Germany). Data were analysed using the Flowing Software 2 (Turku Center for Biotechnology, University of Turku, Finland). Senescent cells were harvested by trypsinization and seeded in microwells (5 × 10^3^/well/100 µL) for the MTT assay and 6-well plates (2 × 10^5^/well/2 mL) for the AV/PI and senescence measurements. The same occurred with cells from an exponentially growing population. Two days later, proliferating and senescent cells were treated with Cur-E, Cur-M or control micelles and, as a control, with the senolytic drug ABT-737 (Sigma-Aldrich, Germany). Cells were further incubated at 37 °C, and the amount of viable cells attached onto the plates was determined by the MTT assay, C12FDG and AV/PI staining as described above

### 2.7. Comet Assays (Single Cell Gel Electrophoresis, SCGE)

To assess the amount of SSBs and DSB, as well as the oxidative DNA damage, alkaline comet assays with and without FPG and neutral comet assays were performed as described [35]. In brief, cells were collected by trypsinization, resuspended in ice-cold PBS (Bio&Sell, Leipzig, Germany) and embedded in ultra-pure low-melting agarose (Invitrogen, Carlsbad, CA, USA. The cell suspension was then evenly spread onto slides that were pre-coated with agarose, dried and submersed in precooled alkaline or neutral lysis buffer (2.5 M NaCl, 100 mM EDTA, 10 µM Tris, 1% sodium lauroyl sarcosinate, 1% Triton X-100, 10% DMSO, pH 10 and 7.5, respectively) for 1 h at 4 °C. For the assay with FPG, slides were pre-incubated in FPG-buffer (40 mM HEPES, 0.5 mM EDTA, 100 mM KCL, 0.2 mg/mL BSA, pH 8) for 5 min after which cells were incubated with 1 µg/mL fapy-DNA glycosylase (FPG) (kind gift from Prof. Epe, Mainz) for 40 min at 37 °C. The slides were then incubated for 20 min in alkaline (300 mM NaOH, 1 mM EDTA, pH >13) or neutral running buffer (90 mM Tris, 90 mM boric acid, 2 mM EDTA, pH 7.5) for denaturation and equilibration. Cells were then electrophoresed with 300 mA for 15 min at 4 °C. Slides from the alkaline comet assay were then washed in neutralization buffer (400 mM Tris, pH 7.5). All slides were rinsed with water, fixed in 100% ethanol for 5 min and dried. For analysis, cells were stained with propidium iodide (Sigma-Aldrich) and evaluated using a fluorescence microscope (Microphot-FXA, Nikon, Tokyo, Japan) and the Comet IV software (Perceptive Imaging, Liverpool, UK). As a positive control, cells were incubated for 30 min with 300 µM tBOOH.

### 2.8. ROS

The induction of ROS was measured by an increase in DCF-fluorescence with the flow cytometer using the FACS Canto II flow cytometer (Becton Dickinson GmbH, Heidelberg, Germany). Cells were incubated for 30 min at 37 °C with 10 µM H_2_DCFDA (Invitrogen, Carlsbad, CA, USA) in serum-free medium (Gibco, Life Technologies Corporation, Paisley, UK). Cells were collected by trypsinization and re-suspended in cold PBS (Bio&Sell, Leipzig, Germany). Fluorescence was measured immediately. As a positive control, cells were incubated for 30 min with 300 µM tert-butyl hydroperoxide (tBOOH).

### 2.9. Statistics

All data are given as the mean with the standard error of the mean (SEM), and differences between group means were statistically evaluated using the Two-Way ANOVA, unpaired Student’s *t*-test or Mann–Whitney test, as appropriate, and considered significant at *p* < 0.05.

## 3. Results

### 3.1. Effect of Cur-E and Cur-M on the Viability of Cells

**Figure 1:**
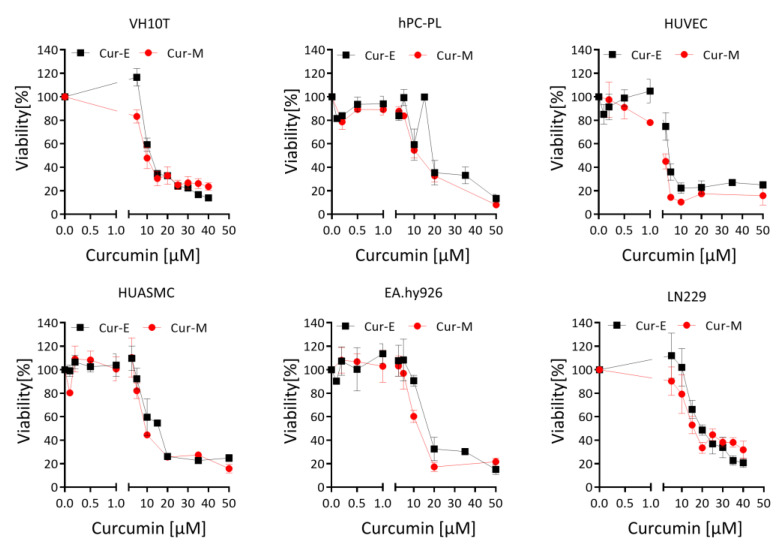
Effect of Cur-E and Cur-M on the viability of different primary cells and the tumor cell line LN229. Dose-dependent toxicity of Cur-E and Cur-M was determined in the MTT assay upon 72 h treatment of LN229, VH10tert, EA.hy926, HUVEC, HUASMC and hPC-PL. Data are the mean +/− SEM of 3 independent experiments.

First, we studied the effect of Cur-E and Cur-M on the cell’s viability, which was comparatively analysed on different cell systems, including human telomerase-immortalized fibroblasts VH10T, cancer cells (the glioblastoma line LN229), human endothelial cells (the line EA.hy926), human primary vascular endothelial cells (HUVEC), human primary smooth muscle cells (HUASM) and human primary pericytes (hPC-PL). As shown in Figure 1, in all cell systems, Cur-E and Cur-M were equally cytotoxic, reducing the viability in a concentration range between 5 and 50 µM. Below 5 µM, neither Cur-E nor Cur-M exerted a significant reduction in viability in all cell types, except HUVEC (for D_50_ doses see Supplementary Materials, Figure S2). Freshly isolated human monocytes, macrophages and T cells responded with curcumin concentrations >15 µM (Supplementary Materials, Figure S3).

### 3.2. Cur-E and Cur-M Induce Cell Death by Apoptosis

**Figure 2:**
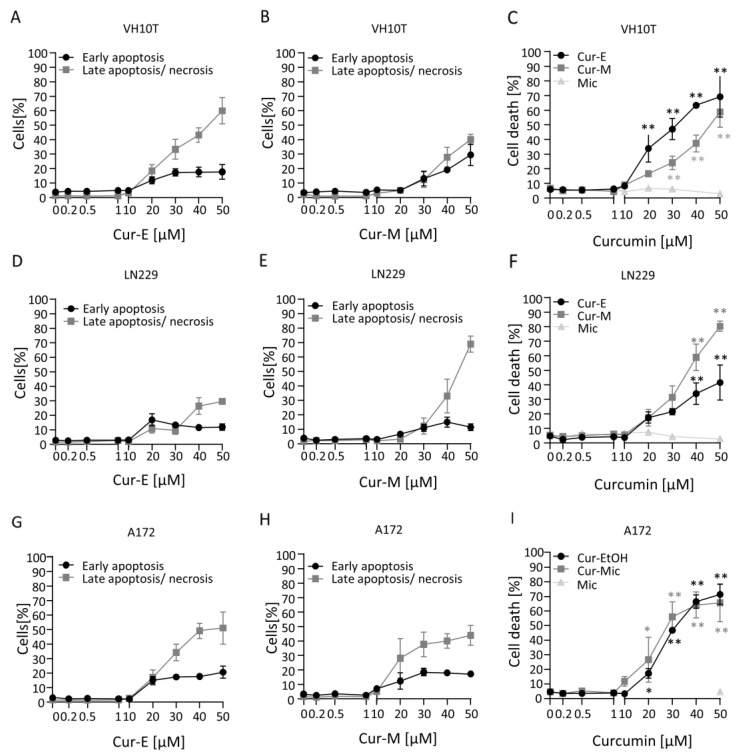
Induction of cell death following curcumin treatment. Proliferating VH10T (**A**–**C**), LN229 (**D**–**F**) and A172 (**G**–**I**) cells were treated with the indicated concentrations of Cur-E or Cur-M for 48 h. Apoptosis, late apoptosis/necrosis and total cell death were measured by AV/PI flow cytometry. Data are the mean of 3 independent experiments ± SEM. Statistical analysis was performed using the Two-way ANOVA. Significant elevation above the control was observed with 20 µM curcumin. Water micelles were ineffective. * *p* < 0.05; ** *p* < 0.01.

The reduction in viability observed in the MTT assay can be due to different mechanisms, including metabolic impairment, arrest of proliferation and genuine cell death. To compare in more detail the cytotoxic potential of the curcumin formulations, we measured apoptosis and necrosis by AV/PI flow cytometry. The data shown in Figure 2 demonstrate that Cur-E and Cur-M are effective in inducing apoptosis. The dose range was similar in human fibroblasts VH10T and the glioblastoma cell lines LN229 and A172. The threshold concentration at which a significant increase was observed was 20 µM, while at 10 µM the levels were still insignificant above the background. In VH10T cells, Cur-E was slightly more effective in inducing cell death than Cur-M. In the glioma cell lines, this difference was not observed. Control micelles (Mic) administered in the same amount did not show any cytotoxic effects (Figure 2). Treatment for 1 h and post-incubation for 48 h was ineffective in inducing apoptosis (Supplementary Materials Figure S4).

### 3.3. Cur-E and Cur-M Have No Senolytic Activity

**Figure 3:**
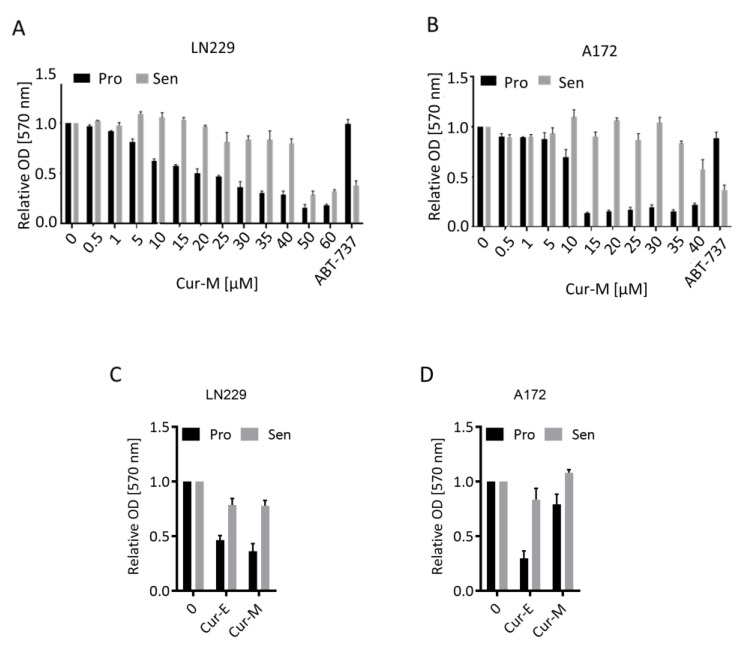
Effect of curcumin on the viability of proliferating and senescent cells. Proliferating (Pro) and senescent (Sen) LN229 (**A**,**C**) and A172 (**B**,**D**) cells were treated with the indicated dosages of Cur-E or Cur-M. (**A**,**B**) Dose response of proliferating and senescent LN229 (**A**) and A172 (**B**) cells upon curcumin treatment. (**C**,**D**) Comparison of Cur-E and Cur-M in reduction in viability in LN229 (**C**) and A172 (**D**) cells. Viability (MTT OD_570_) of non-treated control was set to 1. The senolytic drug ABT-737 served as a positive control.

Many genotoxic agents induce not only apoptosis, but also cellular senescence (CSEN). A potential for curcumin to induce CSEN was shown previously (for a review, see [36]). Here, we pursued to determine the senolytic potential of curcumin, i.e., the selective ability of a compound to kill senescent cells. The methylating anticancer drug temozolomide is a potent inducer of CSEN in p53 functionally active glioblastoma cells [37]. We induced CSEN in LN229 and A172 cells as previously described [38] and assessed whether curcumin is able to kill preferentially senescent cells. For comparison, we used the well-known senolytic drug ABT-737 [39]. We compared the effect of Cur-M on proliferating versus senescent cells. The data revealed that Cur-M was clearly more effective in reducing the viability of proliferating than senescent LN229 (Figure 3A) and A172 (Figure 3B) cells. This is in sharp contrast to ABT-737, which showed a clear senolytic effect on glioblastoma cells, being more effective on senescent cells (Figure 3A,B). The data were substantiated comparing Cur-E and Cur-M, which were equally effective in reducing the viability of proliferating versus senescent cells (Figure 3C,D).

**Figure 4:**
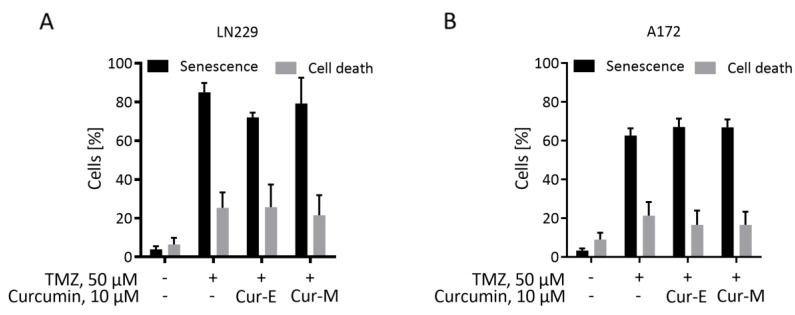
Curcumin does neither reduce the senescence level nor induces apoptosis in a senescent population. Temozolomide-induced senescent LN229 (**A**) and A172 (**B**) cells were treated with Cur-E and Cur-M in a concentration that was not apoptosis-inducing on proliferating cells. Senescence and cell death were measured by C12FDG and AV/PI staining, respectively, in a flow cytometer. Mean ± SEM, statistical analysis through Two-way ANOVA test. Differences between control, Cur-E and Cur-M were not significant.

To substantiate the data further, we measured the relative amount of senescent and apoptotic cells in the population eight days after temozolomide treatment and, following addition of Cur-E or Cur-M to the medium, two days later. The data revealed that curcumin (10 µM) had no impact on the yield of temozolomide-induced senescent cells and the total cell death (early and late apoptosis/necrosis) level in the population (Figure 4A,B). Overall, the data show that curcumin in a subtoxic concentration has no senolytic activity and does not impact the senescence level, irrespective of whether it is administered as Cur-E or Cur-M.

### 3.4. Analysis of Genotoxic Effects of Curcumin

**Figure 5:**
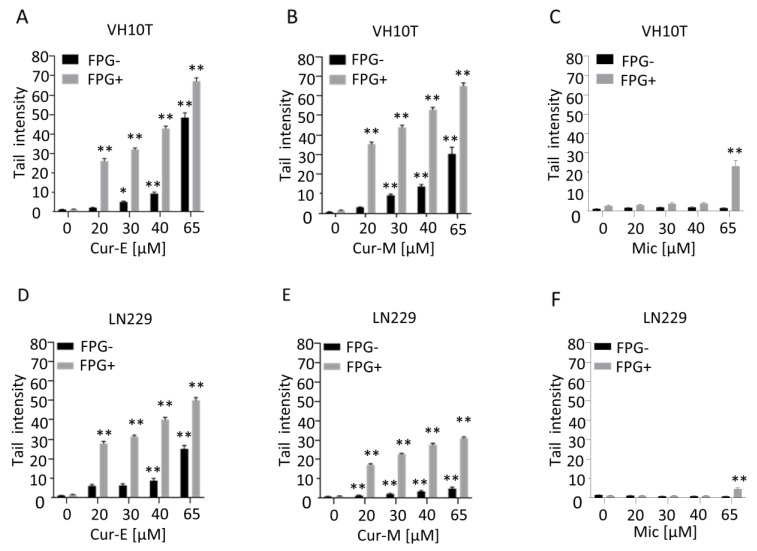
Curcumin induces DNA damage in a dose-dependent manner. Proliferating VH10T (**A**–**C**) and LN229 (**D**–**F**) cells were treated with the respective concentrations of curcumin. (**A**,**D**), curcumin was solubilised in ethanol (Cur-E) or (**B**,**E**), administered as micelles (Cur-M). As control, cells were also treated with water-micelles (Mic, **C**,**F**). Alkaline comet assay with and without FPG was performed 24 h post-treatment. Asterisks indicate significant difference to control * *p* < 0.05; ** *p* < 0.01.

The observation that curcumin is able to induce ROS in human cells ([29,40] and Supplementary Materials Figure S5) prompted us to study the genotoxic potential of the compound, focusing on a comparison of Cur-E and Cur-M. First, we measured the amount of DNA damage in human VH10T fibroblasts as a function of the concentration of curcumin after 24 h of exposure of exponentially growing cells. We used the alkaline comet assay, which measures mostly single-strand breaks, and the FPG-modified alkaline assay, which detects FPG-cleavage sites such as 8-oxo-G [41]. As shown in Figure 5, curcumin was positive in the alkaline comet assay at concentrations >20 µM. In the more sensitive FPG-comet assay, already 20 µM curcumin provoked a significant effect in all cell lines. There was no difference between Cur-E and Cur-M (Figure 5A,B), and control micelles (Mic) did not display any genotoxic effect (Figure 5C and Supplementary Materials Figure S6). Similar data were obtained with LN229 cells upon treatment with Cur-E, Cur-M and control micelles (Figure 5D–F). In a low concentration range between 0.2 and 5 µM, curcumin did not induce strand-breaks, as determined in the alkaline comet assay (Supplementary Materials Figure S7).

**Figure 6:**
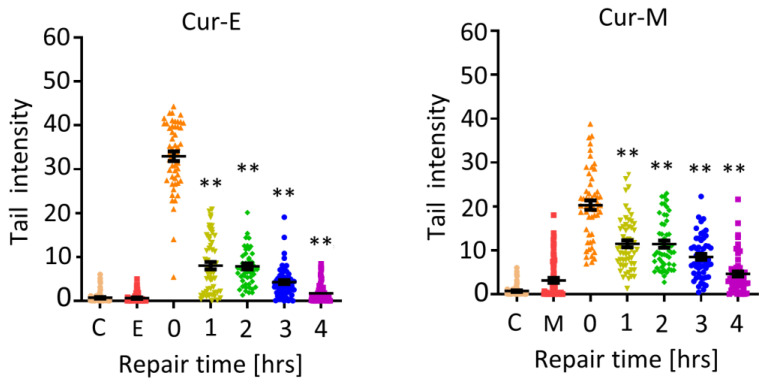
Induction and repair of DNA damage following treatment of VH10T cells with Cur-E and Cur-M. C, untreated control; E, ethanol control; M, micelles control; treatment with 40 µM occurred for 1 h (repair time 0 h) and post-incubation for 1, 2, 3 and 4 h. Statistical analysis was performed using the Two-way ANOVA. ** *p* < 0.01.

Curcumin was positive in the alkaline comet assay already after short-term exposure. Thus, after 1 h of treatment, effects were observed, irrespective of whether treatment occurred with Cur-E or Cur-M (Figure 6; repair time zero). This was confirmed in a repair experiment, where VH10T cells were treated with curcumin (40 µM) for 1 h and post-incubated for 1, 2, 3 and 4 h before harvest. The data show that repair of damage clearly occurred in the 4 h post-treatment period. Again, there was no clear difference between Cur-E and Cur-M (Figure 6), and micelles without curcumin were without any effect (Figure 6). In sum, the data revealed that Cur-E and Cur-M bear genotoxic potential at a high dose level (≥20 µM). The lesions appear to be short lived and are subject to repair.

**Figure 7:**
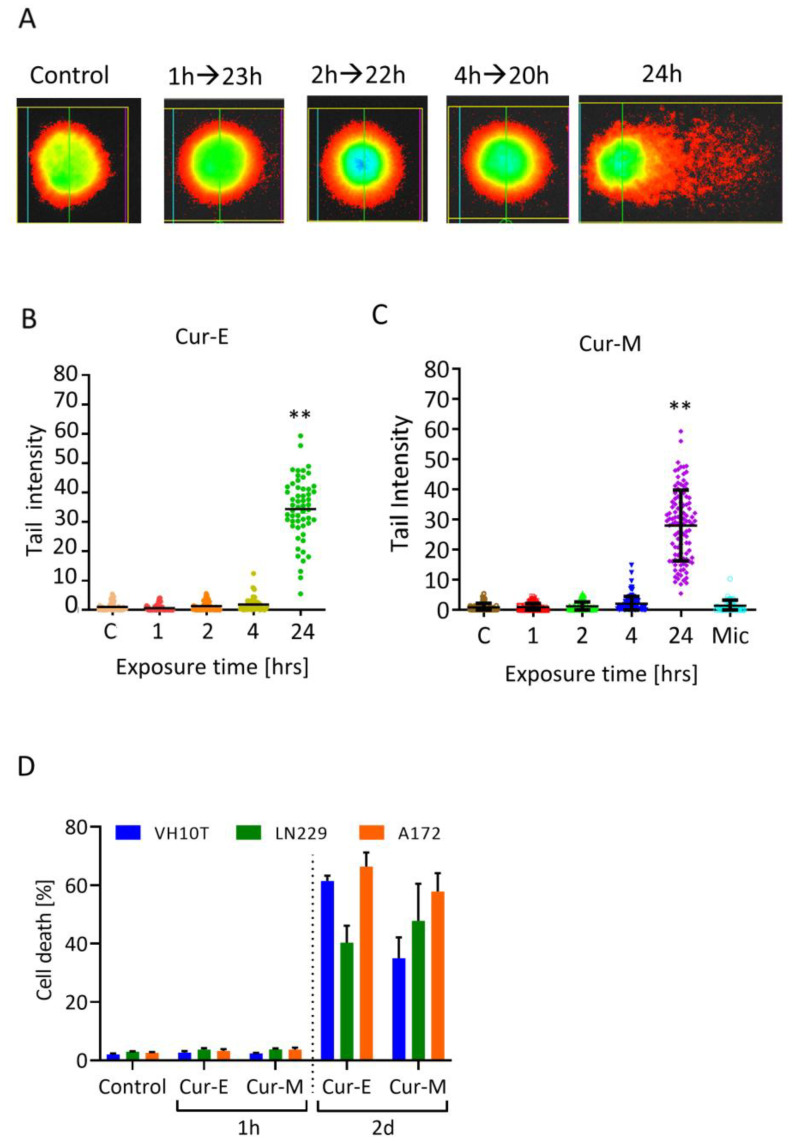
Effect of Cur-E and Cur-M in short- and long-term exposure experiments. (**A**) Representative images of stained single cells. (**B**,**C**) Effect of Cur-E and Cur-M in VH10T cells following exposure for the indicated times and post-exposure. Harvest occurred for all treatments 24 h after the onset of treatment with 40 µM curcumin. FPG comet assay. Statistical analysis was performed using the Two-way ANOVA. (**D**) Death (apoptosis, necrosis) of VH10T, LN229 and A172 cells following treatment of exponentially growing populations with Cur-E or Cur-M (40 µM) for 1 h and post-incubated 47 h in curcumin free medium or treated for the whole period of 48 h, until cell harvest. Cells were measured by AV/PI flow cytometry. Data are the mean of 3 experiments ± SEM. ** *p* < 0.01.

The finding of short-lived lesions indicates that curcumin has to be present in the medium in order to elicit a genotoxic effect in the long-term setting. This was proven by an experiment in which cells were treated for 1, 2, 4 and 24 h followed by a recovery time of 23, 22, 20 and 0 h, respectively. Cells were harvested after a total incubation time of 24 h and analysed by the alkaline comet assay. As shown in Figure 7A (for representative images) and Figure 7B,C (for quantification), treatment for 1, 2 and 4 h did not give rise to DNA damage compared to treatment over the whole incubation period of 24 h. This supports the notion that the lesions are short-lived and subject to repair. In order to provoke long-term effects, it is obviously necessary that curcumin is permanently present in the medium. This is especially the case for the observed toxic effects; short-term treatment (1 h) even at a high dose of curcumin did not result in apoptosis induction while long-term exposure (48 h) did (Figure 7D).

**Figure 8:**
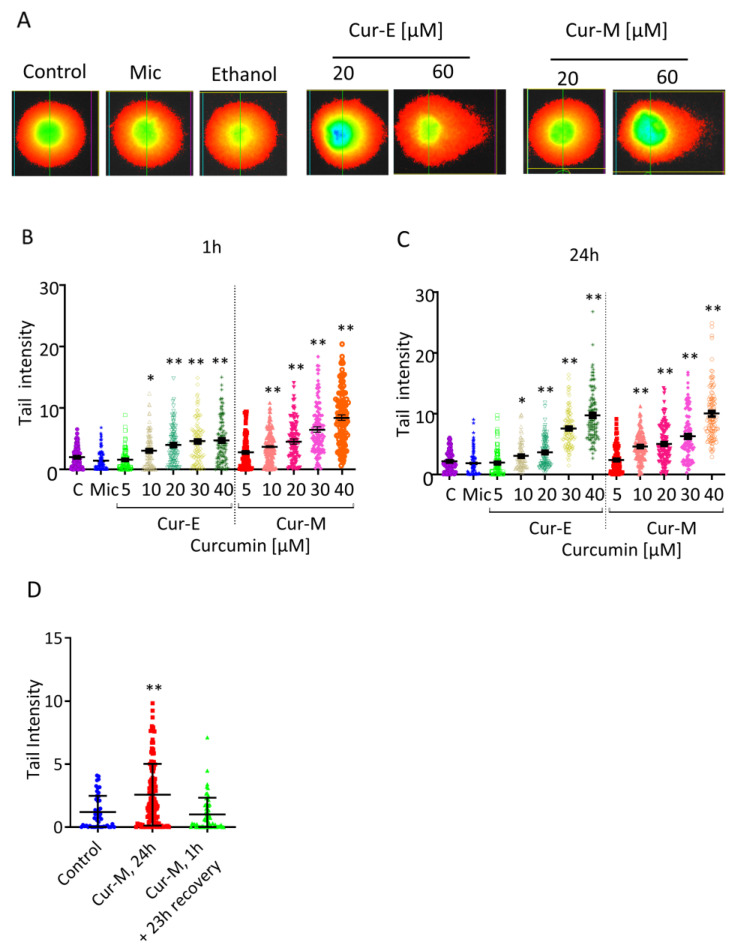
Effect of Cur-E and Cur-M on VH10T cells measured in the neutral comet assay. (**A**) Examples of stained cells following single cell gel electrophoresis (24 h treatment). (**B**) Quantification upon 1 h treatment. (**C**) Quantification upon 24 h treatment. (**D**) Cells were not treated (control) or treated for 1 h and post-incubated for 23 h (Cur-M, 1 h) or for 24 h (Cur-M, 24 h) with Cur-M. Then, 100 cells were evaluated per treatment level. C, non-treated control; Mic, micelles only corresponding to 40 µM. Statistical analysis was performed using the Two-way ANOVA test. * *p* < 0.05; ** *p* < 0.01.

To elucidate whether curcumin bears a potential to induce DSBs, we made use of the neutral comet assay (SCGE, representative pictures Figure 8A). Treatment for 1 h (Figure 8B) or 24 h (Figure 8C) resulted in a dose-dependent increase in tail intensity, and the lowest effective dose was 10 µM curcumin. Again, Cur-E and Cur-M were equally effective in inducing effects in the neutral SCGE, and micelles without curcumin (Mic) were ineffective (Figure 8B,C). Treatment with a concentration of 40 µM curcumin for 1 h and a recovery of 23 h did not result in significant DSBs, while treatment over a 24 h period resulted in a significant yield of DSBs (Figure 8D). These data indicate recovery of cells through repair of DSBs that were induced with a high curcumin concentration during a 1 h exposure period.

## 4. Discussion

Curcumin has been used as a food ingredient in Asia for thousands of years and is also extensively applied in traditional Chinese medicine (phytomedicine), especially for the treatment of inflammation-related and neurodegenerative diseases [7,8]. Today, it is widely used worldwide as a spice and food additive (E100). Native curcumin has a low bioavailability [15,16] due to its poor water solubility and rapid metabolism in the liver [17,18]. It is soluble in an alkaline environment (although unstable under these conditions) and in ethanol and dimethyl sulfoxide (DMSO), but these are solvents that are suitable for experimental purposes only [19]. When preparing dishes containing curcumin (e.g., “curry”), turmeric is usually suspended in oil under heat. From this suspension, in which micellar structures form, curcumin can better be absorbed in the gastrointestinal tract. To overcome uptake limitations, a formulation of curcumin embedded in micelles on PEG80 basis was developed and shown to have superb uptake properties [21,22,23,42,43,44,45]. Micellar curcumin was recently also tested in a feeding study and demonstrated to be safe and effective as an anticancer agent in a colorectal mouse model [46].

Although curcumin solubilised in DMSO was extensively studied (for a review, see [14]), comparative analyses of curcumin administered as micelles and solubilised in an organic solvent are lacking. Thus, it is unclear whether micellar curcumin and the micelles itself have properties that are different from curcumin solubilised in an organic solvent. To provide an answer to this question, we rigorously compared Cur-E and Cur-M as to their cytotoxic, senolytic and genotoxic effects on normal and cancer cells in vitro.

First, we show that Cur-E and Cur-M reduce the viability of human cells irrespective of its origin, including human diploid fibroblasts, human primary pericytes, endothelian cells, smooth muscle cells, established endothel cells and human glioblastoma cells. In these cell models, the concentration range in which a decline in viability was observed was 10–60 µM, and Cur-E and Cur-M caused a similar response. The cytotoxic effect was confirmed showing that Cur-E and Cur-M induce cell death by apoptosis in human fibroblasts and cancer (glioblastoma) cells. Apoptosis increased dose-dependently, and the lowest concentration inducing a significant effect was 20 µM (48 h treatment). Again, Cur-E and Cur-M were similarly effective.

Curcumin-induced apoptosis was reported in various cell systems, including NIH3T3 and L929 mouse fibroblasts, human colon carcinoma cells (HT-29), human breast carcinoma cells (MCF-7) and rat glioma cells (C-6)] [28,47,48,49,50]. Induction of apoptosis was preceded by a ROS burst, which followed the activation of the mitochondrial apoptosis pathway through a release of AIF and cytochrome C from the mitochondria, activation of caspases and PARP-1 cleavage (as an indicator of apoptosis). The p53-p21-CDC2 signaling pathway was also shown to be activated, leading to cell cycle arrest via Rb dephosphorylation and downregulation of cyclin D1 and cyclin D3 [50]. It is interesting that, in all studies, the induction of apoptosis occurred in a narrow dose range of 10–80 µM curcumin (dissolved in DMSO). Apoptotic effects occurred in MCF-7 cells as early as 24 h after treatment with curcumin and reached a maximum 48 h after administration [50]. Cytotoxicity is therefore an immediate, acute effect of curcumin. Our data are in line with this. Irrespective of the cell type, cell death by apoptosis occurred with an exposure concentration of >10 µM. This was the case in a variety of human primary cells and cancer cell lines. According to our data, 10 µM can be considered as a cytotoxic threshold dose for human cells, which applies for both Cur-E and Cur-M.

Curcumin was shown to be able to induce cellular senescence in different cell systems [31,32,51]. It is unclear, however, whether it bears senolytic activity, which is defined as killing senescent, but not proliferating, cells. To our knowledge, there is no data to show that curcumin is able to kill especially cancer cells in which senescence was induced following chemotherapy. We tested this assumption and found that Cur-E and Cur-M killed proliferating cells more efficiently than senescent cells. This is in sharp contrast to ABT-737, which is a well-known senolytic agent [39] and used as a positive control in our experiments. Our data suggest that curcumin, irrespective of the formulation, is not a senolytic agent. We are aware of the limitations of this study as we assessed the effect in only two cancer cell lines and under limited treatment conditions. More detailed studies are clearly required in order to come to a more generalized conclusion (see also [36]).

The finding that curcumin is able to induce ROS (our data and [40]) prompted us to investigate in detail the question of DNA damage induction. Treatment with Cur-E and Cur-M induced in human fibroblasts dose-dependently DNA breaks which was most obvious in the FPG-modified comet assay, in which ROS-induced DNA damage, such as 8-oxo-G, is recognized by the FAPY-DNA glycosylase and converted into single-strand breaks that can be detected in the assay. The alkaline comet assay was also positive without FPG; however, higher curcumin concentrations were required to elicit the same effect. The observed effects are not a byproduct of cytotoxicity, as positive effects in the FPG comet assay can be observed already after 1 h of exposure. This indicates that oxidative DNA damage is induced immediately after curcumin treatment, which is in line with a previous report [40]. It is important to note that removal of Cur-E or Cur-M from the medium returned the DNA tail moment to the basal level, indicating efficient repair of DNA lesions. Thus, short-term treatment did not result in permanent DNA damage, indicating the effects are transitory and not stable. The finding indicates that, in assessing the genotoxic potential, it is important to take into consideration not only the exposure concentration, but also the period of exposure. This applies also to the endpoint apoptosis, which was measured 2 days after addition of Cur-E or Cur-M to the medium. The continuous presence of curcumin in the medium was necessary for eliciting a significant toxic and genotoxic effect on cells.

To determine whether curcumin has the potency to induce DSBs, we made use of the neutral comet assay. In this assay, curcumin was weakly positive with a concentration ≥10 µM. Similar effects were observed when cells were treated for 1 or 24 h, and a recovery period after a 1 h treatment resulted in their complete disappearance. The effects measured in the neutral comet assay might be due to overlapping ROS-induced single-strand breaks, overlapping single-strand breaks and base-excision repair patches, or they represent directly induced DSBs. The effect was also transient; it vanished after post-exposure recovery of cells in the absence of curcumin. It should be noted that curcumin was reported to be negative in the γH2AX foci assay [52].

Curcumin is negative in bacterial test systems [25] and not mutagenic. It is considered an anti-oxidant in the low dose range, causing protection against some genotoxins, e.g., radiation-induced DNA damage [27]. However, in the high dose range (>5 µM), curcumin acts as a radical generator and pro-oxidant, causing a ROS burst and the associated oxidative DNA damage [40]. Thus, our data are in line with a report showing that curcumin induces DNA strand breaks and 8-oxo-guanine in HepG2 cells at concentrations >2.5 µg/mL (6.8 µM) [29]. In the concentration range of 2.5–10 µg/mL, curcumin induced micronuclei in rat PC12 cells [30], likely resulting from chromosome breaks. A study on human peripheral lymphocytes showed that curcumin does not induce sister-chromatid exchanges (SCEs), which are a highly sensitive indicator of DNA damage. However, chromosomal acentric fragments were detected in the high concentration range (50 µg/mL = 135 µM). Interestingly, no chromatid-type aberrations (chromatid gaps, breaks and translocations) that are typical for chemical genotoxins were observed [53]. Since curcumin induces neither direct genotoxic DNA damage (adducts) nor SCEs, it is reasonable to conclude that the observed clastogenicity is based on S-phase independent effects. Curcumin administered at high and cytotoxic concentrations (>10 µM) can therefore be regarded as a substance with a weak radiomimetic activity, at least in cell culture models. Overall, the genotoxic effects observed can all be explained by the intracellular ROS induction after a high dose of curcumin.

It is important to point out that the concentration range in which cytotoxic and genotoxic effects occur is nearly identical in all cell systems used. The overlapping dose range of cytotoxicity and genotoxicity (between 10 and 80 µM continuous treatment) implicates that cells harboring chromosome damage will be eliminated by apoptosis or necrosis. Under these circumstances, it is unlikely that curcumin-induced genotoxic changes will cause long-term effects. Actually, long-term feeding experiments with native curcumin and micellar curcumin did not provide evidence for adverse effects on rodents [54]. The frequently reported tumor-suppressing effect of curcumin [55] supports the view that the cytotoxic and genotoxic effects observed in the micromolar concentration range in vitro are irrelevant under nutritional conditions. On the other hand, they could become important if curcumin is administered locally at a very high, cytotoxic dose level (>20 µM target concentration) in a therapeutic setting, since a toxic effect on tumor cells was sought. An investigation of various cell lines showed that apoptosis induction by curcumin occurred preferentially in transformed, but not primary, cell lines, from which it was concluded that curcumin has a selectively toxic effect on tumor cells. Our data do not confirm this notion, as curcumin was nearly equally toxic in human diploid fibroblasts, primary endothel and smooth muscle cells, monocytes, macrophages and glioblastoma cancer cells.

In summary, micellar curcumin is taken up efficiently into cells, which makes it ideally suitable for experimental purposes since organic solvents can be avoided. Curcumin administered in a micellar formulation had a cytotoxic and genotoxic profile similar to curcumin dissolved in ethanol (similar data were obtained with curcumin dissolved in DMSO). Thus, the data demonstrate that micellar curcumin does not bear toxic and genotoxic properties that are different from native curcumin. From the available data, we define a cytotoxic and genotoxic threshold for curcumin at a concentration of 10 µM, which seems to be independent of the formulation and cell system (our data and data in the literature). Micelles without curcumin were completely devoid of cytotoxic and genotoxic activity. Therefore, micelles, which have a nanosized structure, can be considered as safe transport vehicles. Following uptake of Cur-M in the acceptable daily intake (ADI) range (max 3 mg/kg BW), the concentration of curcumin in the blood serum of humans was estimated to be lower than 100 nM (ref. [56] and J. Frank, personal communication). This is far below the cytotoxic and genotoxic level observed in vitro. It would be interesting to see whether beneficial effects of curcumin, e.g., anti-inflammatory responses, are evoked at curcumin concentrations that are below the toxic threshold.

It is interesting to note that weak genotoxic effects associated with ROS production have been described also for other phytochemicals. Thus, many natural products induce mild oxidative stress in cells [57,58], which may evoke a hormetic cellular stress response, i.e., cellular adaptation leading to protection against challenging genotoxic stress [59]. A main product of ROS in the DNA is 8-OxoG, which appears to be induced at high dose levels (>10 µM) by curcumin [29]. Although 8-oxo-G is a mispairing lesion, recent studies showed that 8-OxoG also acts as a gene regulator. It activates signaling pathways, which may contribute to the beneficial effects described above [60].

## 5. Conclusions

Overall, curcumin reduces cell viability and induces apoptosis as well as genotoxicity in a narrow, overlapping concentration range between 10 and 60 µM. For inducing cytotoxicity through apoptosis, long-term treatment is required; short-term exposure (60 min) was insufficient to elicit toxic effects. Genotoxic effects, measured in the alkaline and comet assay, vanished after post-exposure of cells in curcumin-free medium, indicating that lesions were transiently induced and repaired. Cur-E and Cur-M did not show senolytic activity on cancer cells, in which senescence was induced by the chemotherapeutic temozolomide. Cur-M was not more effective than Cur-E in inducing DNA damage, as measured in comet assays, and micelles were completely devoid of these effects, indicating that micelles do not bear a cytotoxic and genotoxic potential. The concentration dependence of cytotoxicity and genotoxicity reported here, together with animal experiments [46], and the record of the long-term and extensive dietary exposure of humans to native curcumin and the absence of any reports of associated toxicity, support the notion that the natural form of curcumin and the micellar formulation do not bear harmful side effects. The normal intake quantities of curcumin and the recommended inclusion levels of supplements are about 100-fold lower than the concentration considered to be toxic in the in vitro assays, indicating a wide margin of safety.

## Data Availability

Data are available upon request.

## Supplementary Materials

The following are available online at https://www.mdpi.com/article/10.3390/nu13072385/s1. Figure S1: Representative plot of cells stained with annexin V and PI. Figure S2: Effect of curcumin solubilized in ethanol (A) and micellar curcumin (B) on the viability of different primary human cell types. Figure S3: Effect of curcumin on freshly isolated human monocytes, macrophages and T cells. Figure S4: Cytotoxicity (apoptosis, necrosis) of VH10T, LN229 and A172 cells following treatment of exponentially growing populations with Cur-E or Cur-M (40 μM) for 1 h and post-incubated 48 h. Cells were harvested and measured by flow cytometry. N=3,median±SEM. Figure S5: Induction of ROS following Curcumin treatment. Figure S6: Effect of micelles filled with water in the FPG comet assay. Figure S7: Effect of Cur-M on VH10T cells in the alkaline comet assay.

## References

[1] Tan W., Lu J., Huang M., Li Y., Chen M., Wu G., Gong J., Zhong Z., Xu Z., Dang Y. (2011). Anti-cancer natural products isolated from chinese medicinal herbs. Chin. Med..

[2] Demain A.L., Vaishnav P. (2010). Natural products for cancer chemotherapy. Microb. Biotechnol..

[3] Frank J., Fukagawa N.K., Bilia A.R., Johnson E.J., Kwon O., Prakash V., Miyazawa T., Clifford M.N., Kay C., Crozier A. (2019). Terms and nomenclature used for plant-derived components in nutrition and related research: Efforts toward harmonization. Nutr. Rev..

[4] Venturelli S., Burkard M., Biendl M., Lauer U.M., Frank J., Busch C. (2016). Prenylated chalcones and flavonoids for the prevention and treatment of cancer. Nutrition.

[5] Burkard M., Leischner C., Lauer U.M., Busch C., Venturelli S., Frank J. (2017). Dietary flavonoids and modulation of natural killer cells: Implications in malignant and viral diseases. J. Nutr. Biochem..

[6] Aggarwal B.B., Harikumar K. (2009). Potential therapeutic effects of curcumin, the anti-inflammatory agent, against neurodegenerative, cardiovascular, pulmonary, metabolic, autoimmune and neoplastic diseases. Int. J. Biochem. Cell Biol..

[7] Ghosh S., Banerjee S., Sil P.C. (2015). The beneficial role of curcumin on inflammation, diabetes and neurodegenerative disease: A recent update. Food Chem. Toxicol..

[8] Shehzad A., Khan S., Lee Y. (2010). Curcumin therapeutic promises and bioavailability in colorectal cancer. Drugs Today.

[9] Shehzad A., Wahid F., Lee Y.S. (2010). Curcumin in Cancer Chemoprevention: Molecular Targets, Pharmacokinetics, Bioavailability, and Clinical Trials. Arch. Pharm..

[10] Kasi P.D., Tamilselvam R., Skalicka-Woźniak K., Nabavi S.M., Daglia M., Bishayee A., Pazoki-Toroudi H. (2016). Molecular targets of curcumin for cancer therapy: An updated review. Tumor Biol..

[11] Bortel N., Armeanu-Ebinger S., Schmid E., Kirchner B., Frank J., Kocher A. (2015). Effects of curcumin in pediatric epithelial liver tumors: Inhibition of tumor growth and alpha-fetoprotein in vitro and in vivo involving the NFkappaB- and the beta-catenin pathways. Oncotarget.

[12] Hsu C.-H., Chuang S.-E., Hergenhahn M., Kuo M.-L., Lin J.-K., Hsieh C.-Y., Cheng A.-L. (2002). Pre-clinical and early-phase clinical studies of curcumin as chemopreventive agent for endemic cancers in Taiwan. Gan Kagaku Ryoho.

[13] Hsu C.-H., Cheng A.-L. (2007). Clinical Studies with Curcumin. Adv. Exp. Med. Biol..

[14] Allegra A., Innao V., Russo S., Gerace D., Alonci A., Musolino C. (2017). Anticancer Activity of Curcumin and Its Analogues: Preclinical and Clinical Studies. Cancer Investig..

[15] Aller L.L. (2019). What about bioavailability of oral curcumin?. Can. Med. Assoc. J..

[16] Garg A.X., Moist L., Pannu N., Tobe S., Walsh M., Weir M. (2019). Bioavailability of oral curcumin. Can. Med. Assoc. J..

[17] Prasad S., Tyagi A.K., Aggarwal B.B. (2014). Recent Developments in Delivery, Bioavailability, Absorption and Metabolism of Curcumin: The Golden Pigment from Golden Spice. Cancer Res. Treat..

[18] Metzler M., Pfeiffer E., Schulz S.I., Dempe J.S. (2012). Curcumin uptake and metabolism. BioFactors.

[19] Tønnesen H.H. (2002). Solubility, chemical and photochemical stability of curcumin in surfactant solutions. Studies of curcumin and curcuminoids, XXVIII. Die Pharm..

[20] Liu W., Zhai Y., Heng X., Che F.Y., Chen W., Sun D., Zhai G. (2016). Oral bioavailability of curcumin: Problems and advancements. J. Drug Target..

[21] Schiborr C., Kocher A., Behnam D., Jandasek J., Toelstede S., Frank J. (2014). The oral bioavailability of curcumin from micronized powder and liquid micelles is significantly increased in healthy humans and differs between sexes. Mol. Nutr. Food Res..

[22] Khayyal M.T., El-Hazek R.M., Elsabbagh W., Frank J., Behnam D., Abdel-Tawab M. (2018). Micellar solubilisation enhances the antiinflammatory activities of curcumin and boswellic acids in rats with adjuvant-induced arthritis. Nutrition.

[23] Kocher A., Bohnert L., Schiborr C., Frank J. (2016). Highly bioavailable micellar curcuminoids accumulate in blood, are safe and do not reduce blood lipids and inflammation markers in moderately hyperlipidemic individuals. Mol. Nutr. Food Res..

[24] Dützmann S., Schiborr C., Kocher A., Pilatus U., Hattingen E., Weissenberger J., Geßler F., Quick-Weller J., Franz K., Seifert V. (2016). Intratumoral Concentrations and Effects of Orally Administered Micellar Curcuminoids in Glioblastoma Patients. Nutr. Cancer.

[25] Damarla S.R., Komma R., Bhatnagar U., Rajesh N., Mulla S.M.A. (2018). An Evaluation of the Genotoxicity and Subchronic Oral Toxicity of Synthetic Curcumin. J. Toxicol..

[26] Decker E.A. (1997). Phenolics: Prooxidants or antioxidants?. Nutr. Rev..

[27] Srinivasan M., Prasad N.R., Menon V.P. (2006). Protective effect of curcumin on gamma-radiation induced DNA damage and lipid peroxidation in cultured human lymphocytes. Mutat. Res..

[28] Seyithanoğlu M.H., Abdallah A., Kitiş S., Guler E.M., Koçyiğit A., Dündar T.T., Papaker M.G. (2019). Investigation of cytotoxic, genotoxic, and apoptotic effects of curcumin on glioma cells. Cell. Mol. Biol..

[29] Cao J., Jia L., Zhou H.-M., Liu Y., Zhong L.-F. (2006). Mitochondrial and Nuclear DNA Damage Induced by Curcumin in Human Hepatoma G2 Cells. Toxicol. Sci..

[30] Mendonça L.M., Dos Santos G.C., Antonucci G.A., Santos A.C., Bianchi M.D.L.P., Antunes L.M.G. (2009). Evaluation of the cytotoxicity and genotoxicity of curcumin in PC12 cells. Mutat. Res. Toxicol. Environ. Mutagen..

[31] Hendrayani S.-F., Al-Khalaf H.H., Aboussekhra A. (2013). Curcumin Triggers p16-Dependent Senescence in Active Breast Cancer-Associated Fibroblasts and Suppresses Their Paracrine Procarcinogenic Effects. Neoplasia.

[32] Grabowska W., Kucharewicz K., Wnuk M., Lewinska A., Suszek M., Przybylska D., Mosieniak G., Sikora E., Bielak-Zmijewska A. (2015). Curcumin induces senescence of primary human cells building the vasculature in a DNA damage and ATM-independent manner. AGE.

[33] He Y., Roos W.P., Wu Q., Hofmann T.G., Kaina B. (2019). The SIAH1–HIPK2–p53ser46 Damage Response Pathway is Involved in Temozolomide-Induced Glioblastoma Cell Death. Mol. Cancer Res..

[34] Kaina B., Beltzig L., Piee-Staffa A., Haas B. (2020). Cytotoxic and Senolytic Effects of Methadone in Combination with Temozolomide in Glioblastoma Cells. Int. J. Mol. Sci..

[35] Nikolova T., Marini F., Kaina B. (2017). Genotoxicity testing: Comparison of the gammaH2AX focus assay with the alkaline and neutral comet assays. Mutat. Res..

[36] Bielak-Zmijewska A., Grabowska W., Ciolko A., Bojko A., Mosieniak G., Bijoch Ł., Sikora E. (2019). The Role of Curcumin in the Modulation of Ageing. Int. J. Mol. Sci..

[37] Knizhnik A.V., Roos W., Nikolova T., Quiros S., Tomaszowski K.-H., Christmann M., Kaina B. (2013). Survival and Death Strategies in Glioma Cells: Autophagy, Senescence and Apoptosis Triggered by a Single Type of Temozolomide-Induced DNA Damage. PLoS ONE.

[38] He Y., Kaina B. (2019). Are There Thresholds in Glioblastoma Cell Death Responses Triggered by Temozolomide?. Int. J. Mol. Sci..

[39] Yosef R., Pilpel N., Tokarsky-Amiel R., Biran A., Ovadya Y., Cohen S., Vadai E., Dassa L., Shahar E., Condiotti R. (2016). Directed elimination of senescent cells by inhibition of BCL-W and BCL-XL. Nat. Commun..

[40] Thayyullathil F., Chathoth S., Hago A., Patel M., Galadari S. (2008). Rapid reactive oxygen species (ROS) generation induced by curcumin leads to caspase-dependent and -independent apoptosis in L929 cells. Free Radic. Biol. Med..

[41] Collins A.R. (2014). Measuring oxidative damage to DNA and its repair with the comet assay. Biochim. Biophys. Acta (BBA) Gen. Subj..

[42] Chen S., Wu J., Tang Q., Xu C., Huang Y., Huang D. (2020). Nano-micelles based on hydroxyethyl starch-curcumin conjugates for improved stability, antioxidant and anticancer activity of curcumin. Carbohydr. Polym..

[43] Sun Y., Li Y., Shen Y., Wang J., Tang J., Zhao Z. (2019). Enhanced oral delivery and anti-gastroesophageal reflux activity of curcumin by binary mixed micelles. Drug Dev. Ind. Pharm..

[44] Karabasz A., Lachowicz D., Karewicz A., Mezyk-Kopec R., Stalińska K., Werner E., Cierniak A., Dyduch G., Bereta J., Bzowska M. (2019). Analysis of toxicity and anticancer activity of micelles of sodium alginate-curcumin. Int. J. Nanomed..

[45] Frank J., Schiborr C., Kocher A., Meins J., Behnam D., Schubert-Zsilavecz M., Abdel-Tawab M. (2017). Transepithelial Transport of Curcumin in Caco-2 Cells Is significantly Enhanced by Micellar Solubilisation. Plant Foods Hum. Nutr..

[46] Seiwert N., Fahrer J., Nagel G., Frank J., Behnam D., Kaina B. (2021). Curcumin Administered as Micellar Solution Suppresses Intestinal Inflammation and Colorectal Carcinogenesis. Nutr. Cancer.

[47] Jiang M., Yang-Yen H., Yen J.J., Lin J. (1996). Curcumin induces apoptosis in immortalized NIH 3T3 and malignant cancer cell lines. Nutr. Cancer.

[48] Kuo M.-L., Huang T.-S., Lin J.-K. (1996). Curcumin, an antioxidant and anti-tumor promoter, induces apoptosis in human leukemia cells. Biochim. Biophys. Acta (BBA) Mol. Basis Dis..

[49] Anto R.J., Mukhopadhyay A., Denning K., Aggarwal B.B. (2002). Curcumin (diferuloylmethane) induces apoptosis through activation of caspase-8, BID cleavage and cytochrome c release: Its suppression by ectopic expression of Bcl-2 and Bcl-xl. Carcinogenesis.

[50] Kizhakkayil J., Thayyullathil F., Chathoth S., Hago A., Patel M., Galadari S. (2010). Modulation of curcumin-induced Akt phosphorylation and apoptosis by PI3K inhibitor in MCF-7 cells. Biochem. Biophys. Res. Commun..

[51] Grabowska W., Mosieniak G., Achtabowska N., Czochara R., Litwinienko G., Bojko A., Sikora E., Bielak-Zmijewska A. (2019). Curcumin induces multiple signaling pathways leading to vascular smooth muscle cell senescence. Biogerontology.

[52] Nikolova T., Dvorak M., Jung F., Adam I., Kramer E., Gerhold-Ay A. (2014). The gammaH2AX assay for genotoxic and nongenotoxic agents: Comparison of H2AX phosphorylation with cell death response. Toxicol. Sci..

[53] Sebastià N., Soriano J., Barquinero J.F., Villaescusa J.I., Almonacid M., Cervera J., Such E., Silla M.A., Montoro A. (2012). In vitro cytogenetic and genotoxic effects of curcumin on human peripheral blood lymphocytes. Food Chem. Toxicol..

[54] Ganiger S., Malleshappa H., Krishnappa H., Rajashekhar G., Rao V.R., Sullivan F. (2007). A two generation reproductive toxicity study with curcumin, turmeric yellow, in Wistar rats. Food Chem. Toxicol..

[55] Unlu A., Nayir E., Kalenderoglu M.D., Kirca O., Ozdogan M. (2016). Curcumin (Turmeric) and cancer. J. BUON.

[56] Eckert G.P., Schiborr C., Hagl S., Abdel-Kader R., Müller W.E., Rimbach G., Frank J. (2013). Curcumin prevents mitochondrial dysfunction in the brain of the senescence-accelerated mouse-prone 8. Neurochem. Int..

[57] Dörsam B., Wu C.-F., Efferth T., Kaina B., Fahrer J. (2014). The eucalyptus oil ingredient 1,8-cineol induces oxidative DNA damage. Arch. Toxicol..

[58] Berdelle N., Nikolova T., Quiros S., Efferth T., Kaina B. (2011). Artesunate Induces Oxidative DNA Damage, Sustained DNA Double-Strand Breaks, and the ATM/ATR Damage Response in Cancer Cells. Mol. Cancer Ther..

[59] Radak Z., Ishihara K., Tekus E., Varga C., Posa A., Balogh L. (2017). Exercise, oxidants, and antioxidants change the shape of the bell-shaped hormesis curve. Redox Biol..

[60] Boldogh I., Hajas G., Aguilera-Aguirre L., Hegde M.L., Radak Z., Bacsi A., Sur S., Hazra T.K., Mitra S. (2012). Activation of Ras Signaling Pathway by 8-Oxoguanine DNA Glycosylase Bound to Its Excision Product, 8-Oxoguanine. J. Biol. Chem..

