# Supplementary material for: Cytotoxic, Genotoxic and Senolytic Potential of Native and Micellar Curcumin
Source: Nutrients. 2021 Jul 13;13(7):2385. doi: 10.3390/nu13072385 (PMC8308652; doi:10.3390/nu13072385)
Supplement: Supplementary file 1 [file nutrients-13-02385-s001.zip › nutrients-1257672-supplementary.pdf]

Supplementary material

Cytotoxic, genotoxic and senolytic potential of native and micellar curcumin

Lea Beltzig, Anna Frumkina, Christian Schwarzenbach and Bernd Kaina*

Institute of Toxicology, University Medical Center, Obere Zahlbacher Straße 67, 55131 Mainz, Germany

*Corresponding author

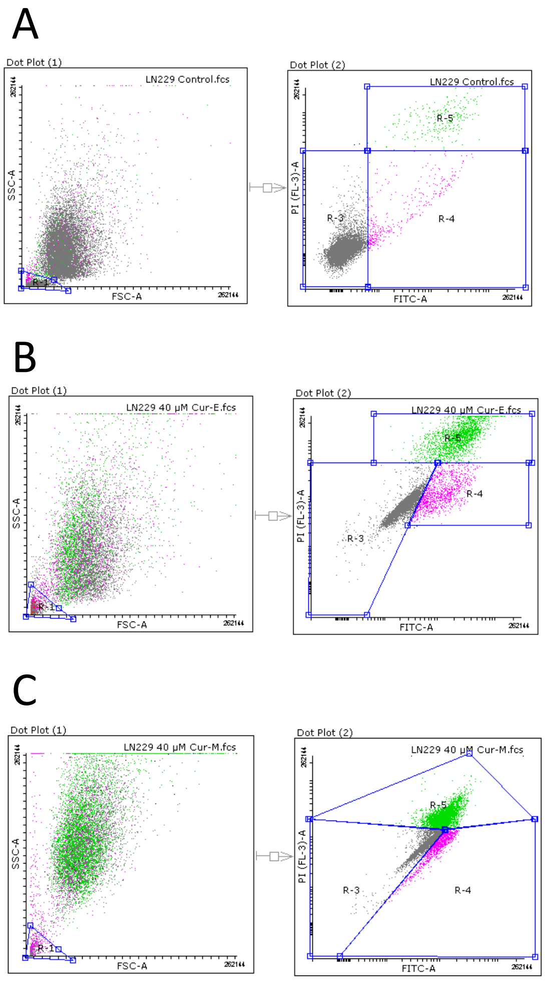

Figure S1: Representative plot of cells stained with annexin V and PI. A, control; B treated with Cur-E; C, treated with Cur-M (40 µM, 48 h curcumin). Lower left corner indicates life cells, lower right the apoptotic and upper right the late apoptotic/necrotic fraction.

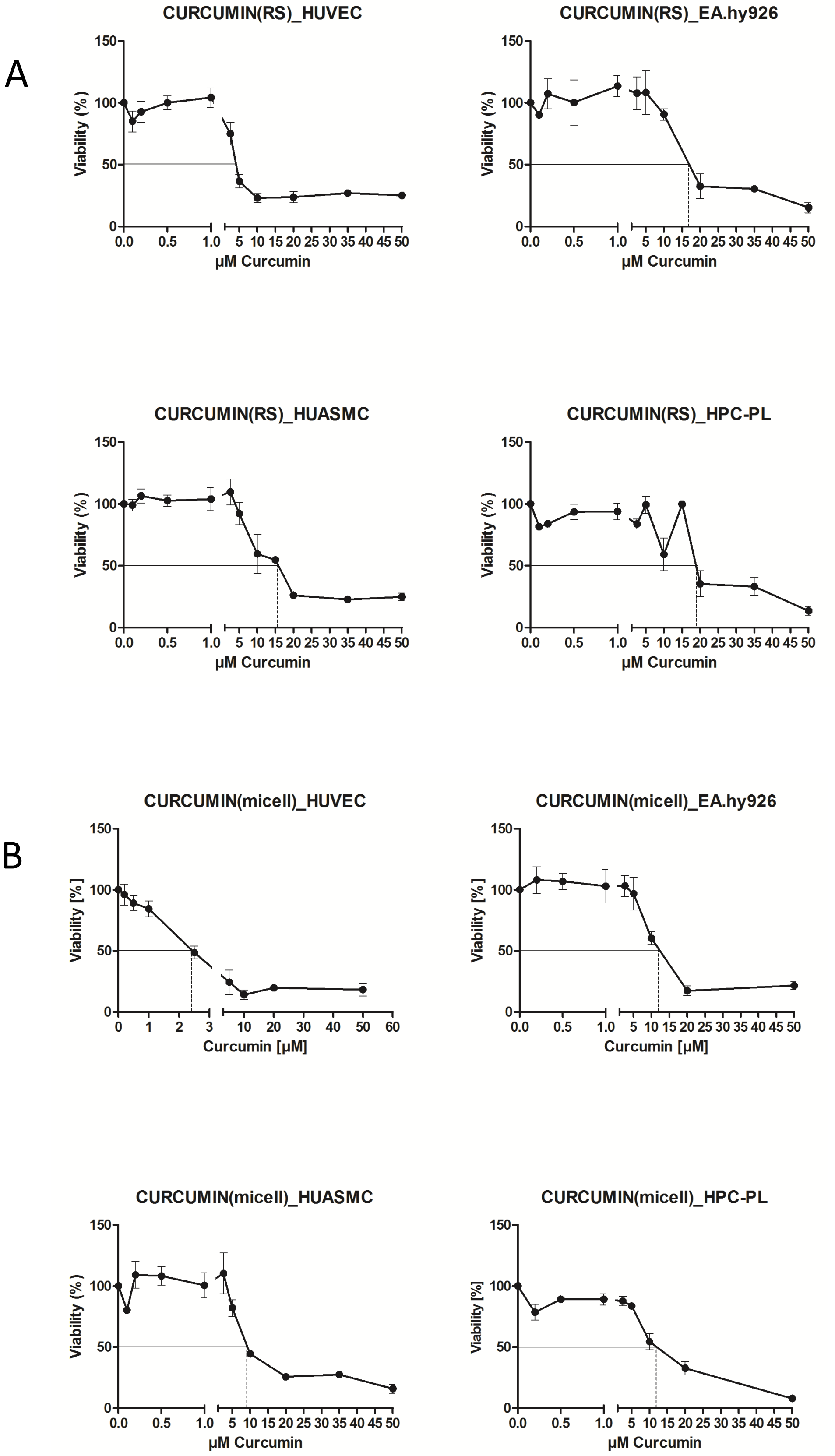

Figure S2: Effect of curcumin solubilized in ethanol (A) and micellar curcumin (B) on the viability of different primary human cell types.

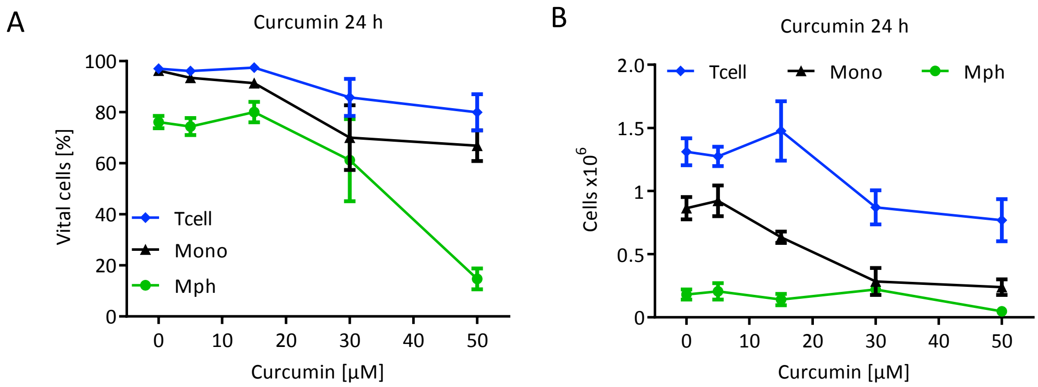

Figure S3: Effect of curcumin on freshly isolated human monocytes, macrophages and T cells. A, percentage of living cells; B, cell number in the population. Cells were incubated for 24 h in the presence of curcumin.

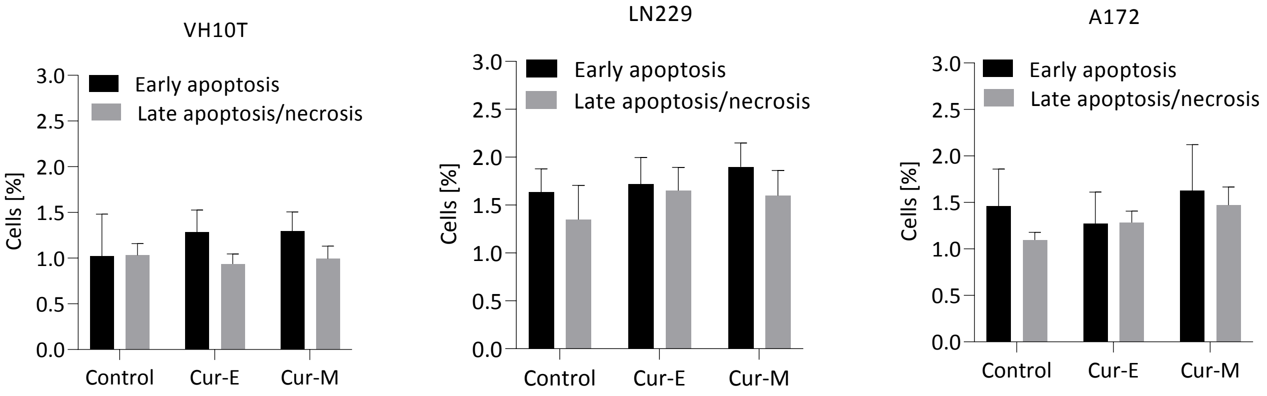

Figure S4: Cytotoxicity (apoptosis, necrosis) of VH10T, LN229 and A172 cells following treatment of exponentially growing populations with Cur-E or Cur-M (40 µM) for 1 h and post-incubated 48 h. Cells were harvested and measured by flow cytometry. N=3, median±SEM

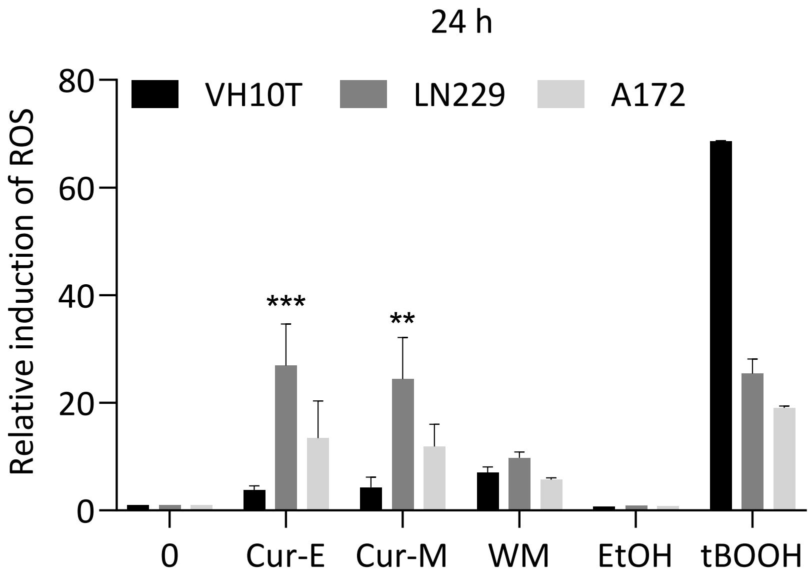

Figure S5: Induction of ROS following Curcumin treatment. Proliferating LN229, A172 and VH10T cells were treated with 40 µM curcumin administered solubilized in ethanol (Cur-E) or packed in micelles (Cur-M). ROS was measured 24 h post treatment via DCFDA staining. Micelles without curcumin, ethanol and tBOOH served as controls.

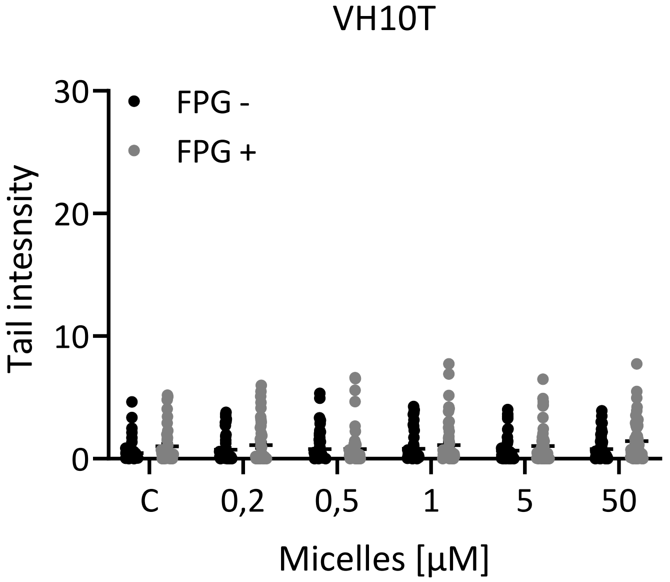

Figure S6: Effect of micelles filled with water in the FPG comet assay. Treatment of VH10T cells occurred for 60 min in complete medium.

A

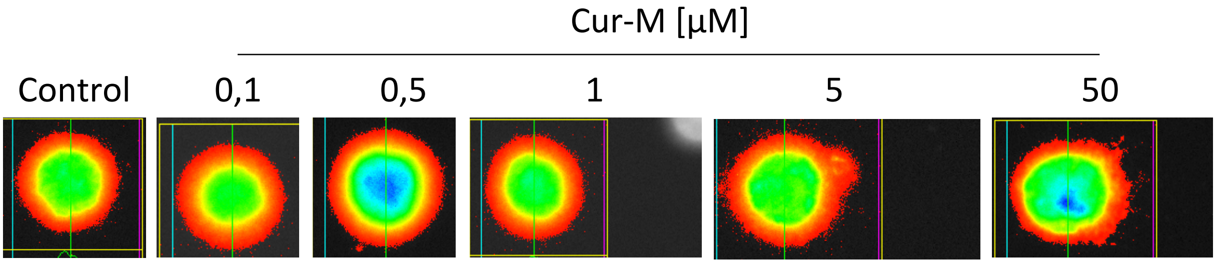

B

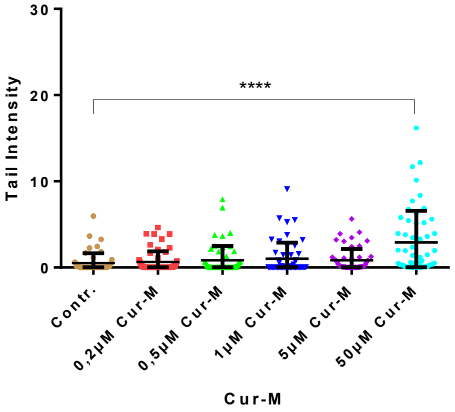

Figure S7: Effect of Cur-M on VH10T cells in the alkaline comet assay.
A, representative examples, B, quantification.
Cur-M was added to the medium 24 h before harvest of cells by trypsinization and commet assay.
